# The Influence of the Combination of Carboxylate and Phosphinate Pendant Arms in 1,4,7-Triazacyclononane-Based Chelators on Their ^68^Ga Labelling Properties

**DOI:** 10.3390/molecules200713112

**Published:** 2015-07-21

**Authors:** Gábor Máté, Jakub Šimeček, Miroslav Pniok, István Kertész, Johannes Notni, Hans-Jürgen Wester, László Galuska, Petr Hermann

**Affiliations:** 1 Department of Nuclear Medicine, Faculty of Medicine, University of Debrecen, Nagyerdei krt 98, H-4032 Debrecen, Hungary; E-Mails: mate.gabor@med.unideb.hu (G.M.); kertesz.istvan@med.unideb.hu (I.K.); galuska.laszlo@med.unideb.hu (L.G.); 2 Lehrstuhl für Pharmazeutische Radiochemie, Technische Universität München, Walther-Meissner-Strasse 3, D-85748 Garching, Germany; E-Mails: jakubsimecek@seznam.cz (J.Š.); johannes.notni@tum.de (J.N.); h.j.wester@tum.de (H.-J.W.); 3 Department of Inorganic Chemistry, Charles University in Prague, Hlavova 2030, 12840 Prague 2, Czech Republic; E-Mail: mirekpniok@gmail.com

**Keywords:** positron emission tomography, metal complexes, macrocyclic ligands, radiopharmaceuticals, tacn derivative, phosphinate complexes, gallium complexes, radiolabelling, PET tracer development, molecular imaging

## Abstract

In order to compare the coordination properties of 1,4,7-triazacyclononane (tacn) derivatives bearing varying numbers of phosphinic/carboxylic acid pendant groups towards ^68^Ga, 1,4,7-triazacyclononane-7-acetic-1,4-bis(methylenephosphinic) acid (NOPA) and 1,4,7-triazacyclononane-4,7-diacetic-1-[methylene(2-carboxyethyl)phosphinic] acid (NO2AP) were synthesized using Mannich reactions with trivalent or pentavalent forms of *H*-phosphinic acids as phosphorus components. Stepwise protonation constants log*K*_1–3_ 12.06, 3.90 and 1.95, and stability constants with Ga^III^ and Cu^II^, log*K*_GaL_ 24.01 and log*K*_CuL_ 16.66, were potentiometrically determined for NOPA. Both ligands were labelled with ^68^Ga and compared with NOTA (tacn-*N*,*N′*,*N″*-triacetic acid) and NOPO, a TRAP-type [tacn-*N*,*N′*,*N″*-tris(methylenephosphinic acid)] chelator. At pH 3, NOPO and NOPA showed higher labelling efficiency (binding with lower ligand excess) at both room temperature and 95 °C, compared to NO2AP and NOTA. Labelling efficiency at pH = 0–3 correlated with a number of phosphinic acid pendants: NOPO >> NOPA > NO2AP >> NOTA; however, it was more apparent at 95 °C than at room temperature. By contrast, NOTA was found to be labelled more efficiently at pH > 4 compared to the ligands with phosphinic acids. Overall, replacement of a single phosphinate donor with a carboxylate does not challenge ^68^Ga labelling of TRAP-type chelators. However, the presence of carboxylates facilitates labelling at neutral or weakly acidic pH.

## 1. Introduction

In analogy to ^99*m*^Tc, the most commonly used radionuclide for single-photon emission tomography (SPECT) [1], the generator-produced radiometal ^68^Ga with its favourable physical properties (89% β^+^-emission; *t*_1/2_ = 67.7 min; *E*_av_(β^+^) = 740 keV) is a valuable resource for decentralised manufacturing of positron emission tomography (PET) radiopharmaceuticals [2,3,4]. For application in nuclear medicine, ^68^Ga is attached to a biological vector as a complex with a suitable chelator that is conjugated to the targeting group, frequently through an additional linker. 

**Figure 1:**
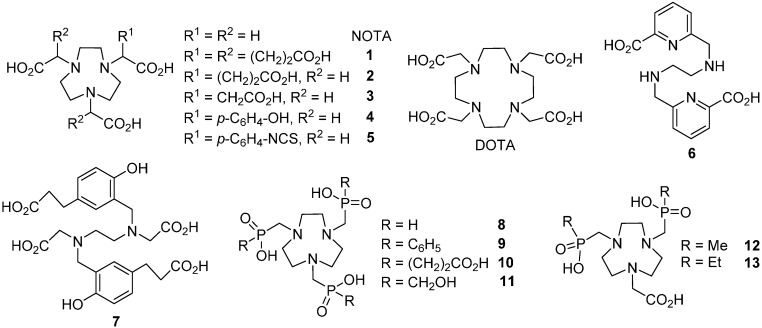
Macrocyclic and open-chain chelators for trivalent gallium.

Current ^68^Ga-based PET is dominated by peptide conjugates of DOTA and NOTA (Figure 1), mainly due to the success of the corresponding radiolabelled octreotide analogues, such as ^68^Ga-DOTATOC, ^68^Ga-DOTATATE, or ^68^Ga-DOTANOC for imaging of neuroendocrine tumours [5,6]. However, although ^68^Ga^3+^ labelling of DOTA is feasible, this chelator has been mainly employed for ^90^Y, ^111^In, ^152^Tb, ^177^Lu, ^212^Pb or ^213^Bi radioisotopes, whose coordination requires higher coordination numbers [7]. Since the coordination chemistry of the radiometal and the chelator determines the labelling conditions [8], an extensive effort has recently been dedicated to the development of improved bifunctional chelators tailored for gallium(III) [9,10,11,12,13,14,15,16,17,18]. For the development of ^68^Ga-based imaging agents, 1,4,7-triazacyclononane-based (tacn-based) NOTA-like bifunctional derivatives (**3** [11], **2** [12], **4** or **5** [13], **1** [14,15]; Figure 1) have been shown as promising chelators for ^68^Ga^3+^ ion. Compared to DOTA, the NOTA-like derivatives can also be labelled efficiently at lower ligand concentrations/excess and lower temperatures [19]. However, ^68^Ga labelling of NOTA proved to be influenced to a considerable extent by metal contaminants present in the ^68^Ge/^68^Ga generator eluates, most notably by Zn^2+^, the inevitable decay product of ^68^Ga [20]. Among the open-chain chelators, despite the lower kinetic inertness of their metal complexes compared to those of macrocyclic ligands, several conjugates of ligands derived from **6** and **7** showed promising results in preclinical and clinical studies [21,22,23].

**Figure 2:**
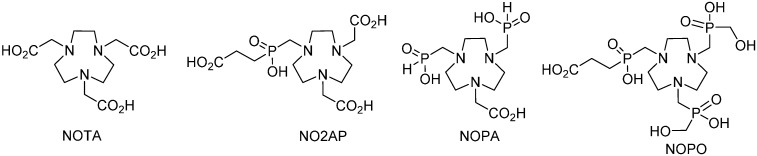
Chelators with acetic/phosphinic acid pendant arms compared in this paper.

Previously, we have evaluated a number of 1,4,7-triazacyclononane-1,4,7-tris(methylenephosphinic acids) (TRAP ligands) for gallium(III) complexation/labelling [9,10,24,25,26]. The phosphinate ligands, **8** [27] and **9** [28], reported earlier, were compared to NOTA, DOTA and phosphinate chelators, **10** and **11** [25]. The TRAP-type chelators showed significantly improved labelling properties when compared with their acetic acid analogues. Apart from the feasibility of labelling at room temperature (RT) and at low chelator concentrations, the higher acidity of phosphinic acids allowed for labelling at acidic conditions (pH < 2), where formation of insoluble ^68^Ga^3+^ hydroxide species is avoided [29]. Among the TRAP chelators, no statistically significant difference in labelling properties has been found; only labelling of the more lipophilic **9** resulted in slightly worse ^68^Ga incorporation efficiency. The TRAP motif was also employed for a straightforward preparation of a PET/MRI bimodal contrast agent, combining TRAP and DOTA structures for Ga^3+^ and Gd^3+^ chelation, respectively [30]. More recently, excellent labelling properties have also been reported for the monoconjugable TRAP-type chelator NOPO [10,31,32] (Figure 2) which combines the pendant arm moieties of **10** and **11**. Interestingly, bringing the asymmetric element to the *N*-substitution pattern did not entail any loss of ^68^Ga-labelling performance. Moreover, NOPO and **10** were found to be highly chemoselective for Ga^3+^, even in the presence of high concentrations of contaminating metallic cations [20].

In order to gain a better understanding of the factors responsible for the ^68^Ga-labelling efficiency of TRAP chelators, we have now investigated two tacn-based bifunctional chelators with asymmetrical *N*-substitution patterns, involving both phosphinate and carboxylate coordination sites (NO2AP and NOPA, Figure 2).

These mixed-donor ligands have been successfully investigated as ligands (e.g., **12** and **13**, Figure 1) selective for Mg^2+^ over Ca^2+^ [33,34]. Their ^68^Ga labelling performance was compared to that of NOTA and NOPO as representatives of symmetrically substituted carboxylate-type and phosphinate-type chelators.

## 2. Results and Discussion

### 2.1. Ligand Synthesis

**Scheme 1:**
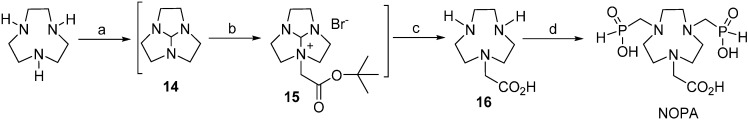
NOPA synthesis. Reagents and conditions: (**a**) (MeO)_2_CHNMe_2_, dioxane, 105 °C, 4 h; (**b**) *t*BuO_2_CCH_2_Br, dioxane, room temp., 1 h; (**c**) NaOH, water/EtOH, reflux, 72 h, 89% based on tacn; (**d**) paraformaldehyde, H_3_PO_2_, water, room temp., 12 h, 63%.

Synthesis of NOPA was carried out according to the reaction sequence shown in Scheme 1. 1,4,7-Triazacyclononane was reacted with *N*,*N*-dimethylformamide dimethyl acetal to give aminal **14** [35] which was monoalkylated *in situ* [36,37], affording the ammonium salt **15** that crystallized from the reaction mixture. This one-pot alkylation followed by hydrolysis is—despite requiring several steps—simple and easy to carry out on a large scale. Compound **16** [33,38,39,40] was then obtained by alkaline hydrolysis of **15**. Moedritzer-Irani (phospho-Mannich) [41] reaction of **15** with phosphinic acid and paraformaldehyde readily afforded NOPA; similarly to the analogous reaction on *N*-monobenzylated tacn [32], the typical formation (according to NMR and MS spectra of the reaction mixture) of *N*-methylated by-products [42] in the last reaction step was suppressed by low reaction temperature. Pure NOPA was obtained in a zwitterionic form after simple purification on a strong cationic exchanger; surprisingly, separation of NOPA from the *N*-methylated by-product on cationic exchange resin was more efficient than that in previously published synthesis of the tris(phosphinic acid) ligand **8** [25].

**Scheme 2:**
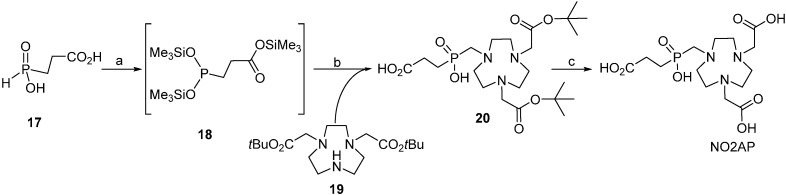
Synthesis of NO2AP through ester **20**. Reagents and Conditions: (**a**) HMDSA, 130 °C, 24 h, quantitative; (**b**) (*i*) paraformaldehyde, HMDSA, 130 °C, 24 h; (*ii*) MeOH, HPLC purification; 46% (based on **19**); (**c**) CF_3_CO_2_H:CH_2_Cl_2_ 1:1, room temp..

Two synthetic pathways were evaluated for the preparation of NO2AP. In the first approach, reaction of the phosphinic acid **17** with tacn-1,7-diacetic acid (NO2A) and formaldehyde in conc. aq. HCl at elevated temperatures (50–70 °C) resulted in the formation of complex mixtures, difficult to separate mainly due to the formation of the *N*-methylated side products. Furthermore, the presence of the free acetic acid pendant arms discourages utilisation of the chelator for selective coupling to a primary amine group in e.g., peptides. Therefore, another route employing a precursor with ester protected *N*-acetates was investigated, in which the phosphite intermediate **18** was generated *in-situ* by reaction of acid **17** with hexamethyldisilazane (HMDSA). The latter intermediate was reacted with tacn-1,7-bis(*t*-butyl acetate) **19** under anhydrous conditions according to our previously reported synthetic procedure [32] to give ester **20** (Scheme 2) [34]. Comparing to the published synthesis (the esterified mixed acetate-phosphinate tacn derivatives have been prepared from the *t*-butyl ester of **16** or from **19** by reaction with paraformaldehyde and MeP(OEt)_2_ or EtP(OEt)_2_, respectively, in anhydrous solvents but the product was isolated in very low overall yields and after difficult purification procedures [34]), the latter procedure is characterized by simple purification and higher overall yield despite the seemingly more demanding synthetic protocols. The silyl groups were removed by treatment with methanol and the free chelator NO2AP was obtained by deprotection with trifluoroacetic acid. The reaction sequence confirmed that silylated phosphites derived from *H*-phosphinic acids are valuable, readily available reagents for the anhydrous variant of Mannich reaction. Utilization of the silylated phosphinic acids for the formation of the >N–CH_2_–P pendant arm might represent a feasible general approach for the synthesis of mixed and/or selectively protected phosphorylated polyazamacrocycles.

### 2.2. Equilibrium Studies

**Figure 3:**
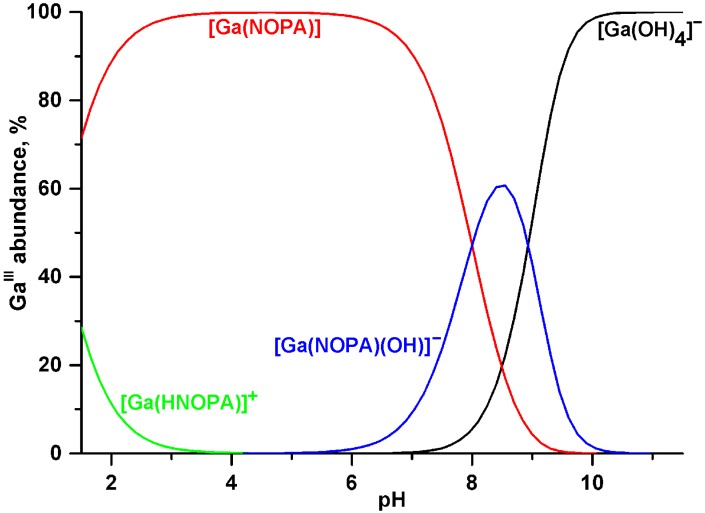
Species distribution diagram of the Ga^3+^-NOPA system.

Protonation constants and gallium(III) complex stability constants of NOPA were determined by potentiometry (Table 1); for the species distribution diagram, see Figure 3. As expected, values of the protonation constants of NOPA were found to be between those of the mother ligands, NOTA and **8**, and, taking into account different experimental conditions, are in a good agreement with the data reported for its methyl- (**12**) and ethyl phosphinate (**13**) analogues (Figure 1) [33,34]. The first protonation constant is relatively high as it should correspond to protonation of the ring amine with the attached acetate moiety, whereas the second protonation constant should be connected with an amine substituted with methyl phosphinate group [34]. Gallium(III) complexation in acidic solution was very fast and complete complex formation was observed at the beginning of titrations at pH 1.5. In this region, formation of a protonated complex was observed (β_HLGa_ = 25.14(8), log*K*_a_ = 1.10). The Ga^3+^ complex stability constant was thus determined through competition with hydroxide anions in alkaline solution. Similarly to other tacn-based ligands [9,25,32], equilibration above pH ~ 6 was slow (more than two weeks) and “out-of-cell” titration method had to be used. Mixed hydroxido species were also found (β_H–1LGa_ = 16.04(5), log*K*_a_ = 8.00). As NOTA derivatives are now commonly used as ligands of choice for complexation of ^64^Cu, stability constants for Cu^2+^-NOPA system were determined as well. The respective complex (β_LCu_ = 16.66(2)) is formed even in very acidic solutions, which nevertheless contained 25% free Cu^2+^ at pH 1.7, enabling the stability constant determination; the chemical model also required a hydroxido species (β_H–1LCu_ = 5.36(2), log*K*_a_ = 11.30). Thermodynamic stabilities of the [Ga(NOPA)] and [Cu(NOPA)]^–^ complexes correlate with the overall ligand basicity [43] (defined as basicity of the ring nitrogen atoms, log*K*_1_ + log*K*_2_) of NOPA and, thus, are between those for the NOTA and **8** complexes.

The protonated [Ga(HNOPA)]^+^ species should be the “*in-cage*” complex as the proton is probably attached to the phosphoryl oxygen atom of the coordinated phosphinate pendant arm [9,25]. Abundance of the [Ga(OH)(NOPA)]^–^ species (Figure 3) is relatively high, and its possible formation during radiolabelling might explain lower radiolabelling yields at higher pH (see below).

**Table 1:** Stepwise protonation (log*K*_n_) and thermodynamic stability (log*K*_GaL_) constants of free ligands and their gallium(III) complexes, respectively (25 °C, *I* = 0.1 M (Me_4_N)Cl). Literature data are given for comparison.

Constant	Ligand					
	NOPA *^a^*	12 *^b^* [34]	8 [25]	10 [15]	NOPO [32]	NOTA [44]
log*K*_1_	12.06	11.7	10.48	11.48	11.96	13.17
	*12.058*(*4*)					
log*K*_2_	3.90	4.24	3.28	5.44	5.22	5.74
	*15.958*(*6*)					
log*K*_3_	1.95	2.10		4.84	3.77	3.22
	*17.910*(*6*)					
log*K*_4_				4.23	1.54	1.96
log*K*_5_				3.45		
log*K*_6_				1.66		
log*K*_GaL_*^c^*	24.04		21.91	26.24	25.0	29.60 [25]
	*24.04*(*6*)					

*^a^* This work; experimentally determined overall protonation/stability constants (logβ*_hlm_*) are in italics; *^b^* 25 °C, *I* = 0.1 M KCl; *^c^* Equilibrium constant for reaction Ga^3+^ + L*^n^*^−^ ↔ [Ga(L)]^(*n*−3)−^ where L*^n^*^−^ is the fully deprotonated ligand.

### 2.3. ^68^Ga Radiolabelling 

**Figure 4:**
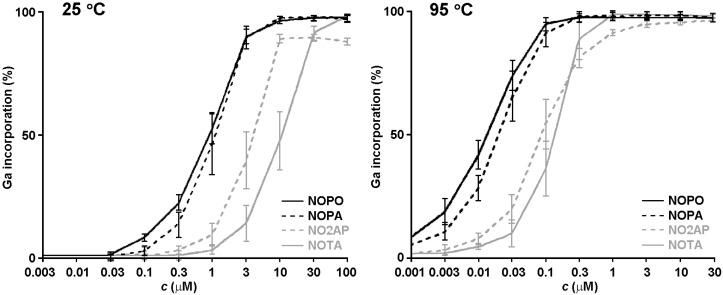
Labelling efficiency of the discussed chelators at 25 and 95 °C at different chelator concentrations (pH = 3, *n* = 3).

Radiolabelling of the chelators at pH 3 exhibited similar shapes and relations of the curves for 95 °C and 25 °C (Figure 4) while, as expected, increased chelator concentrations were required for labelling at ambient temperature. In all cases, the tris(phosphinate) ligand NOPO showed superior labelling compared to the mixed-pendant arm ligands and NOTA. Interestingly, the presence of a single carboxylate donor in NOPA did not significantly affect the labelling performance at pH 3 in comparison to NOPO. Likewise, the behaviour of the monophosphinate ligand NO2AP closely resembled that of NOTA at 95 °C. However at 25 °C, NO2AP showed slightly improved labelling efficiency compared to that of NOTA, although more than 90% radiolabelling yield was not reached, even at fairly high concentrations. Hence, in terms of chelator concentration required for ^68^Ga labelling, the largest difference is observed between the chelators possessing one and two carboxylates or phosphinates. At both temperatures investigated, NOPA could be labelled with three-times better efficiency than NO2AP (comparing at 50% activity incorporation), while NOPO and NOTA are separated by a factor of ten. In addition, the data for NOPO showed a much better reproducibility than those for the other ligands. All this indicates that no less than three phosphinate donors are required to observe high indifference of the TRAP ligand to non-Ga^3+^ ions in the labelling solution, rooted in the exceptional gallium(III) selectivity.

**Figure 5:**
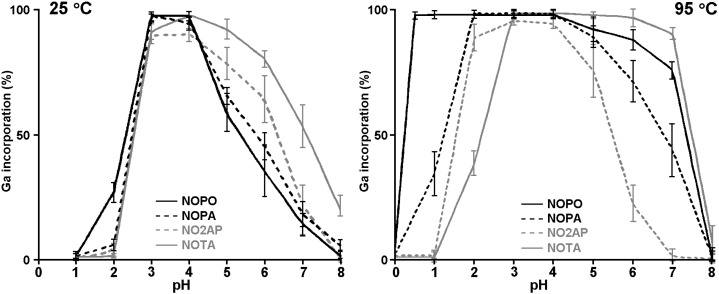
The ^68^Ga activity incorporation into the discussed chelators at 25 °C and 95 °C at different pH values at constant ligand concentrations of 30 and 3 µM, respectively (*n* = 3).

Since all the investigated compounds showed almost quantitative radiolabelling at 3 µM (95 °C) and 30 µM (25 °C), those concentrations were selected for further investigation of labelling efficiency at various pH (Figure 5). At 95 °C, an increasing number of phosphinate side arms mainly resulted in higher labelling yields at lower pH due to the high acidity of phosphinic acids. In accordance with previous results [19], NOPO could be labelled quantitatively already at pH 0.5 and even to a small extent at pH 0. In turn, NOTA showed better performance in the neutral and mildly acidic region. Above pH 8, none of the compounds was labelled anymore.

At ambient temperature, labelling of all chelators was restricted to a much narrower pH region. While NOPO still performed slightly better at lower pH, NOPO, NOPA and NO2AP reached their optimum between pH 3 and 4. However, ^68^Ga incorporation by the latter ligand again did not exceed 90%, while the first two ligands were labelled quantitatively. Above pH 4, labelling efficiency of NOPO was decreasing to a larger extent than that observed for the other chelators. By contrast, and similarly to the situation observed at 95 °C, NOTA performed better than the other ligands between pH 4 and 7, with an optimum at pH 4. Notably, some radioactivity can be clearly incorporated by NOTA even at pH 8.

Overall, radiolabelling results are in line with the previously obtained data on TRAP ligands. Due to the selectivity of phosphinate-containing tacn derivatives for gallium(III) [10,19,20], a lower ligand excess is required for efficient radiolabelling with an increasing number of phosphinate pendant arms. A similar decrease in ^68^Ga incorporation due to presence of the acetate pendant arms has been very recently observed for a diacetate-phosphinate tacn derivative with the *P*-bound –CH_2_CH(PO_3_H_2_)_2_ group [45]. More phosphinate pendant arms also means a better incorporation of ^68^Ga in more acidic solutions due to the higher acidity of phosphinic acids. On the other hand, ligands with more acetate pendant arms are more suitable for ^68^Ga labelling at pH > 4–5. This might be caused by competition with the hydroxide anion, which is more pronounced for complexes exhibiting lower overall thermodynamic stability [25], *i.e.*, for the phosphinate-containing tacn derivatives (see e.g., Figure 3) than for all-carboxylate NOTA.

## 3. Experimental Section

### 3.1. General Information

NOPO [10] and NOTA [46] were synthesized by a published procedure. Ester **19** and 1,4,7-triazacyclononane (tacn) were purchased from CheMatech (Dijon, France). Characterization NMR spectra were recorded using Bruker (600 MHz), Varian UNITY Inova (400 MHz) or VNMRS (300 MHz) spectrometers. ^1^H- and ^13^C-NMR chemical shifts were referenced to *t*-BuOH as internal standard, and ^31^P-NMR chemical shifts were referenced relative to 85% aq. H_3_PO_4_ as external standard. Electrospray mass spectra (ES-MS) spectra were measured with Varian Ion-trap 500 spectrometer in negative or positive modes. High-resolution mass spectra (HR-MS) were measured on UPLC/MS system consisting of Accela 1250 quaternary gradient pump coupled to LTQ Velos Pro/Orbitrap ELITE mass spectrometer (both Thermo, Waltham, MA, USA); samples were dissolved 50% aq. MeOH. Analytical experiments were performed on a HPLC system composed of a Beta 10 gradient pump (ECOM, Prague, Czech Republic) equipped with an active mixer Knauer A0285 and a Topaz dual-UV detector (ECOM), and on Luna RP8, 5 μm, 150 × 4.6-mm column (Phenomenex, Torrance, CA, USA) equipped with a Security Guard system (Phenomenex) holding a C8-cartridge. The mobile phase was continuously vacuum-degassed in a DG 3014 degasser (ECOM, Czech Republic). Semi-preparative HPLC was run with LCD 50K gradient pump (ECOM) and UV-Vis detector LCD2083 (ECOM) on a Luna RP8, 10 μm, 250 × 21.2-mm column (Phenomenex). For the radiolabelling studies, Ultrapur^®^ water, HCl and NaOH were obtained from Merck KGaA (Darmstadt, Germany); all other materials used were commercially available and of analytical grade. At all cases, incorporation of ^68^Ga was determined by radio-TLC on silica-impregnated chromatography paper (Agilent, Santa Clara, CA, USA) with 1 M aq. NH_4_OAc:MeOH 1:1 as mobile phase; scanning and evaluation were performed with a MiniGITA Star TLC-scanner (Raytest, Straubenhardt, Germany).

### 3.2. Syntheses

#### 3.2.1. Synthesis of (1,4,7-Triazacyclononan-1-yl)acetic Acid (**16**)

Tacn (4.00 g, 31 mmol) was dissolved in dioxane (30 mL) and *N*,*N*-dimethylformamide dimethyl acetal (4.40 g, 36.9 mmol) was added. The mixture was heated at 105 °C (in bath) for 4 h, then cooled to room temperature, and *t*-butyl bromoacetate (7.24 g, 37.1 mmol) was added dropwise. Immediately formed suspension was diluted by addition of dioxane (10 mL) and stirred at room temperature for 1 h. Diethyl ether (20 mL) was added and yellow microcrystalline solid was filtered off, washed with Et_2_O and dissolved in solution of NaOH (5.00 g, 125 mmol) in 50% aq. EtOH (40 mL). The solution was refluxed for 72 h, then evaporated to dryness in vacuum and the residue was purified on Dowex 50 in H^+^-form (column size ~3 × 20 cm). The column was washed with water and the product was eluted by 5% aq. NH_3_. The fraction containing pure product was evaporated. The residue was dissolved in water (50 mL) and evaporated in vacuum to dryness; the procedure was repeated twice. The product was isolated as yellow oil (5.20 g, 89%) which solidified upon standing at 4 °C. ^1^H-NMR (300 MHz, D_2_O): δ (ppm) 2.82–2.95 (m, HO_2_CCH_2_NCH_2_CH_2_NH, 8H), 3.11 (s, HNCH_2_CH_2_NH, 4H), 3.30 (s, CH_2_CO_2_H, 2H). ^13^C{^1^H} NMR (75.4 MHZ, D_2_O): δ (ppm) 43.88, 43.98, 50.09 (s 3×, ring CH_2_), 58.26 (s, NCH_2_CO_2_H), 180.44 (s, CO_2_H). MS (ESI, positive mode, *m/z*): 188.3 [M + H]^+^. calc. for M (C_8_H_17_N_3_O_2_) 187.2.

#### 3.2.2. Synthesis of 1,4,7-Triazacyclononane-7-(carboxymethyl)-1,4-bis(methylenephosphinic acid) (NOPA)

Compound **16** (6.20 g, 33.2 mmol) was dissolved in 50% aq. H_3_PO_2_ (36.3 mL, 33.2 mmol) and paraformaldehyde (1.96 g, 65.3 mmol) was added. The mixture in a closed flask was stirred at room temperature for 12 h and paraformaldehyde slowly dissolved. The mixture was evaporated in vacuum to dryness, dissolved in small amount of water and the solution was soaked on Dowex 50 in H^+^-form (column size ~3 × 20 cm). The column was eluted by water and the first acidic fraction, containing phosphinic acid, was discarded, and the product was eluted in further neutral fractions. The fractions containing pure product were collected, evaporated in vacuum and finally freeze-dried to give transparent solid of NOPA (7.20 g, 63%). ^1^H-NMR (300 MHz, D_2_O): δ 3.34 (d, ^2^*J*_PH_ = 9.9 Hz, NCH_2_P, 4H), 3.39–3.56 (m, ring CH_2_, 8H), 3.62 (s, ring CH_2_, 4H), 3.91 (s, NCH_2_CO_2_H, 2H), 7.23 (d, ^2^*J*_PH_ = 546 Hz, PH, 2H). ^13^C{^1^H} NMR (75 MHz, D_2_O): δ 49.97 (s, ring CH_2_), 51.83 (d, ^3^*J*_PC_ = 5.0 Hz, ring CH_2_), 52.07 (d, ^3^*J*_PC_ = 3.8 Hz, ring CH_2_), 56.35 (s, NCH_2_CO_2_H), 56.21 (d, ^2^*J*_PC_ = 88.0 Hz, NCH_2_P), 172.33 (s, CO_2_H). ^31^P-NMR (121 MHz, D_2_O): δ 16.76 (d, ^1^*J*_PH_ = 542 Hz). MS (ESI, positive, *m*/*z*): 366.6 [M + Na]^+^, 344.0 [M + H]^+^; calc. for M (C_10_H_23_N_3_O_6_P_2_) 342.8. HR-MS (positive mode, *m*/*z*): 344.1143 [M + H]^+^, calc. for C_10_H_23_N_3_O_6_P_2_: 343.1062.

#### 3.2.3. Synthesis of 1,4,7-Triazacyclononane-4,7-bis(*t*-butyloxycarbonylmethyl)-1-[methylene(2-carboxyethyl)phosphinic acid] (**20**)

(2-Carboxyethyl)phosphinic acid **17** (0.260 g, 1.9 mmol) [9,47] was dissolved in hexamethyl-disilazane (HMDS, 5 mL) in dry glassware under argon and the solution was heated at 140 °C (in oil bath) for 24 h to give intermediate **18**. Ester **19** (0.200 g, 0.56 mmol) was separately dissolved in HMDS (7 mL) and added into the cooled solution of **18**. Dried paraformaldehyde (0.050 g, 1.6 mmol) was added in one portion, flask was tightly closed and the reaction mixture was heated at 130 °C (in oil bath) for 24 h and then cooled to 25 °C. MeOH (5 mL) was slowly added to remove the trimethylsilyl groups. The reaction mixture was evaporated in vacuum to yield a yellow oil. It was divided into 200 mg portions and each portion was dissolved in water (1 mL), solution was filtered through a 0.5-μm syringe filter and purified using semi-preparative HPLC in gradient mode using solution A (20% MeCN, 20% 0.1 M aq. NH_4_OAc and 60% H_2_O) and B (33% MeCN, 20% 0.1 M aq. NH_4_OAc and 47% H_2_O); flow rate 20 mL/min, gradient: 100% of A to 100% of B in 19 min. The fraction containing pure product (*r*_t_ = 5.7 min) was collected, evaporated in vacuum and finally freeze-dried. Yield 0.130 g (46%, based on *t*Bu_2_NO2A). ^1^H-NMR (600 MHz, D_2_O): δ (ppm) 1.49 (s, CH_3_, 18H), 1.87 (m, PCH_2_CH_2_, 2H), 2.41 (m, PCH_2_CH_2_, 2H), 2.89 (bs, ring CH_2_, 4H), 3.30 (d, ^2^*J*_PH_=7.5 Hz, NCH_2_P, 2H), 3.12 (bs, ring CH_2_, 4H), 3.35 (bs, ring CH_2_, 4H), 3.63 (s, NCH_2_CO, 4H). ^13^C{^1^H} NMR (150 MHz, D_2_O): δ (ppm) 27.63 (d, ^1^*J*_PC_ = 72.0 Hz, PCH_2_CH_2_), 28.03 (s, CH_3_), 30.0 (d, ^2^*J*_PC_ = 3.0 Hz, PCH_2_CH_2_), 47.56 (s, ring CH_2_), 49.66 (s, ring CH_2_), 53.30 (s, ring CH_2_), 53.77 (d, ^1^*J*_PC_ = 88.0 Hz, NCH_2_P), 56.62 (s, NCH_2_CO), 84.27 (s, C_q_), 172.98 (s, NCH_2_CO), 181.29 (d, ^3^*J*_PC_ = 16.7 Hz, PCH_2_CH_2_CO_2_H). ^31^P{^1^H} NMR (121 MHz, D_2_O): δ (ppm) 32.42 (s). MS (ESI, positive, *m*/*z*): 508.3 [M + H]^+^, calc. for M (C_22_H_42_N_3_O_8_P) 507.6. HR-MS (positive mode, *m*/*z*): 508.2797 [M + H]^+^, calc. for C_22_H_42_N_3_O_8_P 507.2710.

#### 3.2.4. Synthesis of 1,4,7-Triazacyclononane-4,7-bis(carboxymethyl)-1-[methylene(2-carboxy-ethyl)phosphinic acid] (NO2AP)

Ester **20** (48.2 mg, 0.095 mmol) was dissolved in dry CH_2_Cl_2_:TFA 1:1 (10 mL) and the solution was stirred in dark at room temperature for 12 h. Solvents were evaporated in vacuum and the crude product was dissolved in water and evaporated, and the procedure was repeated twice. The residue was dissolved in water and the solution was freeze-dried. Product yield 37.1 mg as the trifluoroacetate salt. ^1^H-NMR (600 MHz, D_2_O): δ (ppm) 2.13 (m, PCH_2_CH_2_, 2H), 2.67 (m, PCH_2_CH_2_, 2H), 3.45 (d, ^2^*J*_PH_ = 5.7 Hz, NCH_2_P, 2H), 3.50–3.56 (m, ring CH_2_, 8H), 3.66 (s, ring CH_2_, 4H), 4.14 (s, NCH_2_CO, 4H). ^13^C{^1^H} NMR (150 MHz, D_2_O): δ (ppm) 24.72 (d, ^1^*J*_PC_ = 92.3 Hz, PCH_2_CH_2_) , 27.01 (s, PCH_2_CH_2_), 51.44 (s, ring CH_2_), 52.19 (s, 2× ring CH_2_), 55.01 (d, ^1^*J*_PC_ = 96.4 Hz, NCH_2_P), 57.39 (s, NCH_2_CO), 116.7 (q, ^1^*J*_CF_ = 290.4 Hz), 163.1 (q, ^2^*J*_CF_ = 36.5 Hz), 170.92 (s, NCH_2_CO), 177.14 (d, ^3^*J*_PC_ = 13.5 Hz, PCH_2_CH_2_CO_2_H). ^31^P{^1^H} NMR (121 MHz, D_2_O): δ (ppm) 43.77 (s). MS (ESI, positive, *m*/*z*): 396.1 [M + H]^+^, calc. for M (C_14_H_26_N_3_O_8_P) 395.3. HR-MS (positive mode, *m*/*z*): 396.1534 [M + H]^+^, calc. for C_14_H_26_N_3_O_8_P: 395.1457.

### 3.3. Potentiometry

Potentiometry was carried out (preparation of stock solutions and chemicals, electrode system calibration, titration procedures, equipment and data treatment) according to the previously published procedures [48]. The Ga(NO_3_)_3_ stock solution contained known amount of HNO_3_ to protect it against hydrolysis. Protonation and stability constants were determined in 0.1 M (NMe_4_)Cl at 25.0 °C and they are concentration constants. Protonation constants of NOPA (*c*_L_ = 0.004 M) and Cu-NOPA stability constants (*c*_L_ = *c*_Cu_ = 0.004 M) were determined by normal (“in-cell”) titrations in pH range 1.6–12 with ≈40 points per titration and four parallel titrations. The stability constants in the Ga^3+^–NOPA system were obtained by “out-of-cell” method as described previously (*c*_L_ = *c*_Ga_ = 0.004 M, pH range 1.5–11.5, 25 points per titration, two parallel titrations, equilibration time three weeks) [10,48]. The titration data were treated with OPIUM [49] program. Stability constants of gallium(III) hydroxide species and p*K*_w_ = 13.81 were taken from literature [50,51]. Throughout the text, the pH means −log[H^+^].

### 3.4. ^68^Ga Labelling

The labelling was done manually according to the procedure described in ref. [19]. Briefly, ^68^Ga was eluted from a SnO_2_-based ^68^Ge/^68^Ga-generator (iTHEMBA Labs, Cape Town, South Africa) with 1 M aq. HCl. A 1250-µL fraction containing the highest activity (≈70 MBq) was collected and buffered with 2-[4-(2-hydroxyethyl)-piperazin-1-yl]ethanesulfonic acid (HEPES; 800 µL, 2.7 M aq.). Aliquots of that solution (90 µL) were added to ligand stock solutions of appropriate concentration (10 µL, pH ≈3.0) and left to incubate at 95 °C or 25 °C for 5 min. For pH dependence experiments, pH was adjusted with aq. HCl and/or aq. NaOH.

## 4. Conclusions

A detailed comparison of a series of four tacn-based chelators with various phosphinic/carboxylic acid substitution patterns provided a better understanding of the structural factors governing metal ion complexation properties of this class of ligands. The presence of at least two phosphinic acid pendant arms is a key to the unique ^68^Ga-labelling properties of TRAP-like chelators. Apparently, one phosphinate coordination site of the TRAP motif can be exchanged with a different donor, e.g. carboxylate, without compromising its affinity to gallium(III). On the other hand, the presence of carboxylate groups facilitates the complex formation at neutral or weakly acidic pH. Overall, our findings help with the fine-tuning of metal-binding properties of the pendant-armed 1,4,7-triazacyclononanes and, thus, provide a strong basis for future rational design of these ligands for medical applications.
